# Rare Systemic Response to Titanium Spinal Fusion Implant: Case Report

**DOI:** 10.7759/cureus.7109

**Published:** 2020-02-26

**Authors:** Wendy S Towers, Khalid Kurtom

**Affiliations:** 1 Neurosurgery, University of Maryland Shore Regional Health, Easton, USA; 2 Neurosurgery, University of Maryland School of Medicine, Baltimore, USA

**Keywords:** auto-immune inflammatory syndrome, spinal implants, titanium hypersensitivity, metal allergy

## Abstract

Neurosurgical patients with titanium spinal implant hypersensitivity can be difficult to diagnosis due to its rarity. Suspicion for titanium allergy is generally localized to the hardware site and may initially be thought to be an infectious process. Patients who report anorexia and fatigue over a long duration after the initial post-operative period may be diagnosed with depression rather than a systemic response to spinal metallic instrumentation. To our knowledge, a systemic titanium hypersensitivity reaction to spinal fixation devices has not been reported in the literature. We offer this report to give spine surgeons additional insight into suspected systemic titanium hypersensitivity symptoms which, if remain unidentified, can severely impair patient outcomes. A 67-year-old female with an unreported nickel allergy developed severe debilitating anorexia and fatigue one month post operatively, secondary to minimally invasive thoracic spinal fixation for T11 burst fracture with disruption of posterior elements. Over a two year period, weight loss reached approximately 25 kilograms with loss of muscle mass and subcutaneous tissue surrounding the spinal implants. The screws and rods were removed to avoid skin erosion. Upon hardware removal, the patient had rapid weight gain, improved stamina and generalized sense of well-being. We recommend the removal of spinal hardware in patients with suspected systemic titanium hypersensitivity reaction.

## Introduction

Spinal titanium implant hypersensitivity is an extremely uncommon condition that can lead to a prolonged recovery period without specific symptoms or clinical signs. Hip replacement metal hypersensitivity is more commonly reported due to the dynamic process of metallic shearing within the ball and socket mechanism which leads to aseptic pain complaints [1]. These metallic hypersensitivity reactions are primarily localized to the implantation site and surrounding tissue [2]. Generally, spine surgeons would consider titanium hypersensitivity very low on the differential diagnosis when localized tissue reaction is not apparent even in cases of known metal allergy. 

Here we describe a case of suspected systemic titanium hypersensitivity presenting with prolonged anorexia and fatigue resulting in failure to thrive. A systemic response to spinal metallic instrumentation has not been reported in the literature to our knowledge. We offer this report to give spine surgeons additional insight into suspected systemic titanium hypersensitivity symptoms which, if remain unidentified, can lead to a severe decline in overall patient health. 

## Case presentation

**Figure 1:**
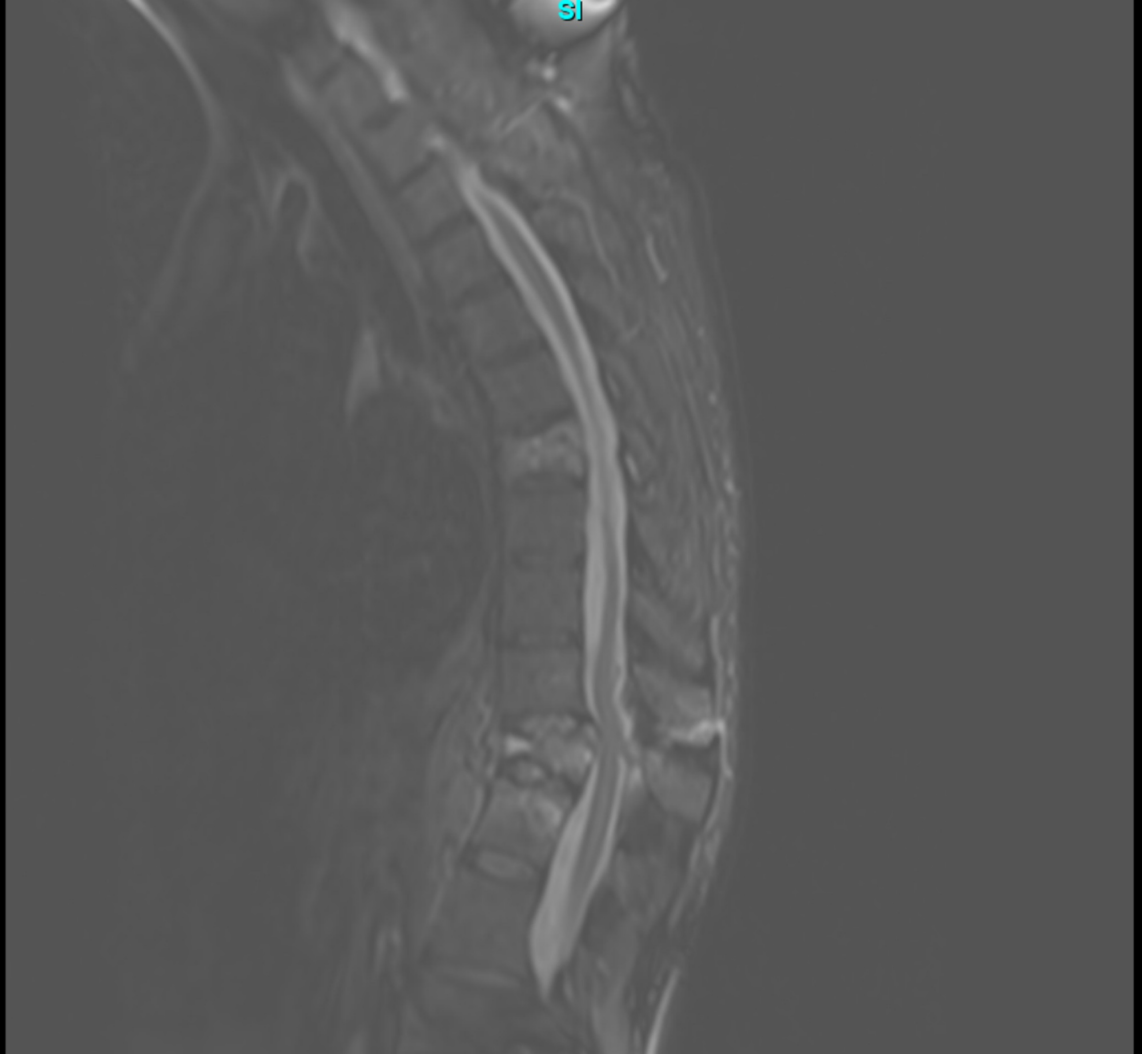
Pre-operative thoracic magnetic resonance imaging (MRI) demonstrating T11 burst fracture

A 67-year-old female with reported allergy to clonazepam and bee venom with a past medical history of aortic valve regurgitation, B12 deficiency, congestive heart failure, coronary artery disease, osteoporosis, and multiple fractures (pelvic, clavicle, ankle) presented with diffuse myelopathy and severe back pain secondary to traumatic T11 burst fracture with disruption of posterior elements including pars and facet joint as well as left clavicle fracture (Figure 1).

**Figure 2:**
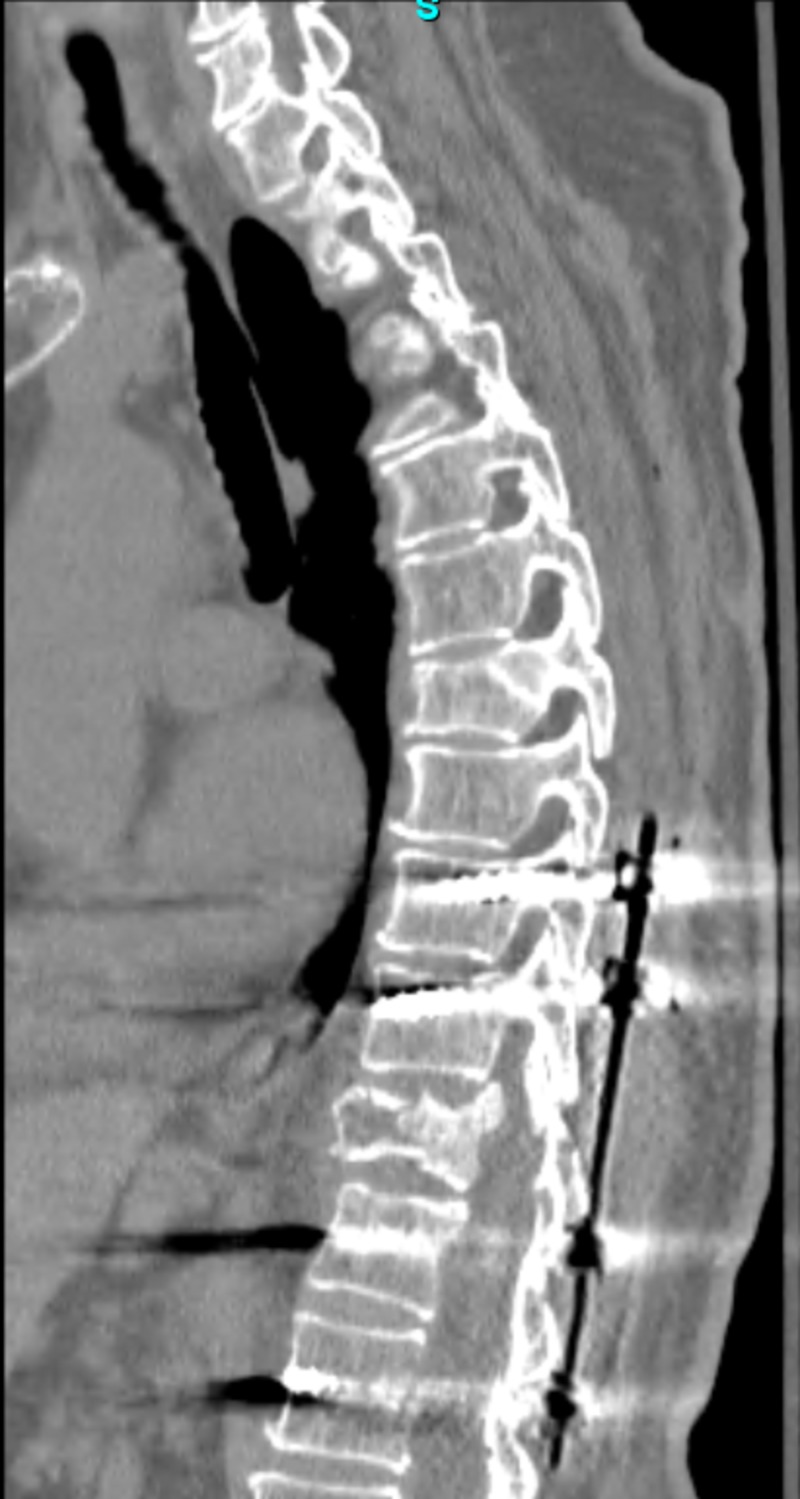
Post-operative computed tomography demonstrating T9-L1 pedicle screw fixation

She underwent T9-L1 minimally invasive bilateral pedicle screw fixation using a titanium fixation system (Figure 2). 

**Figure 3:**
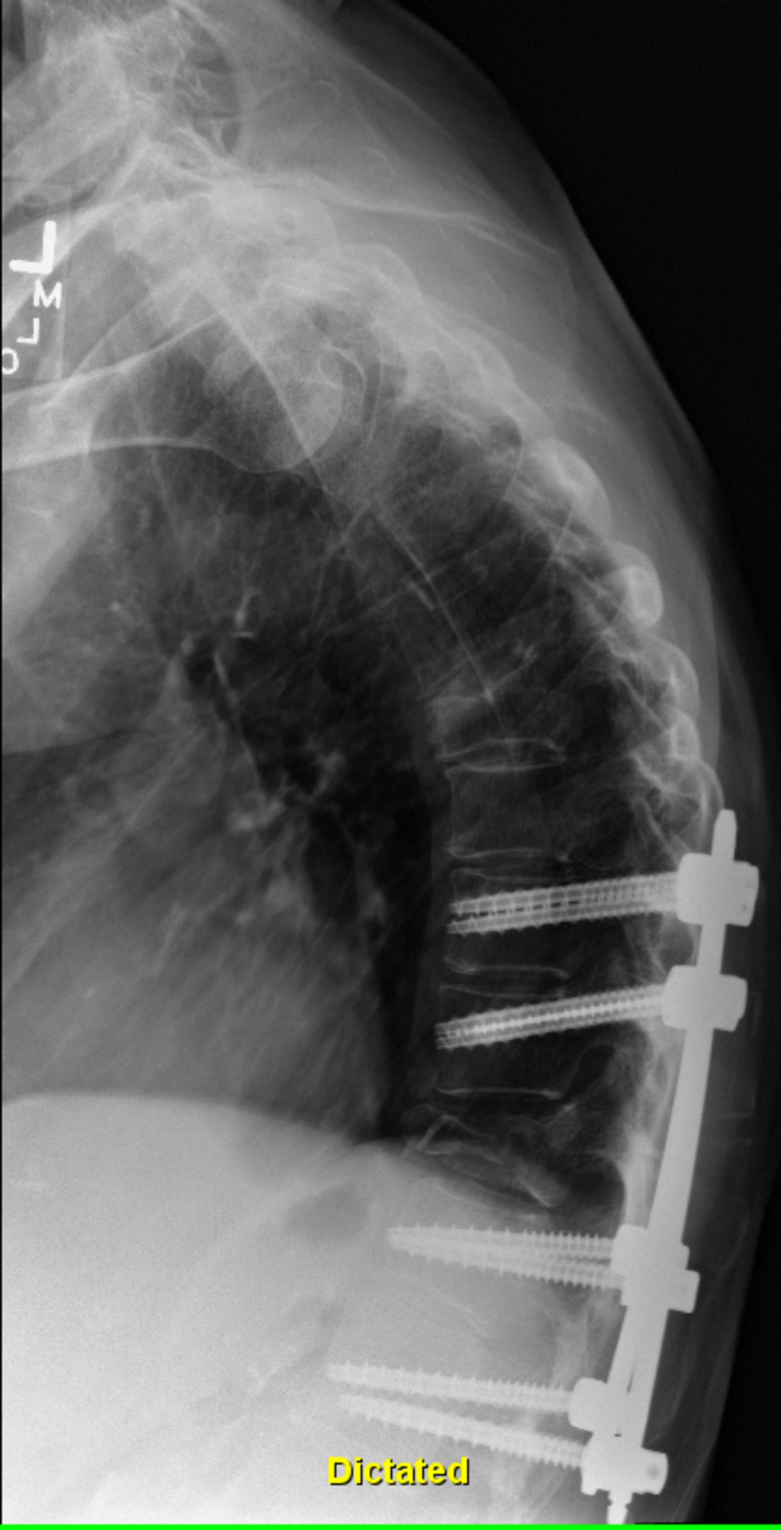
Three months post-operative thoracic spine X-ray with intact hardware without lucency

**Figure 4:**
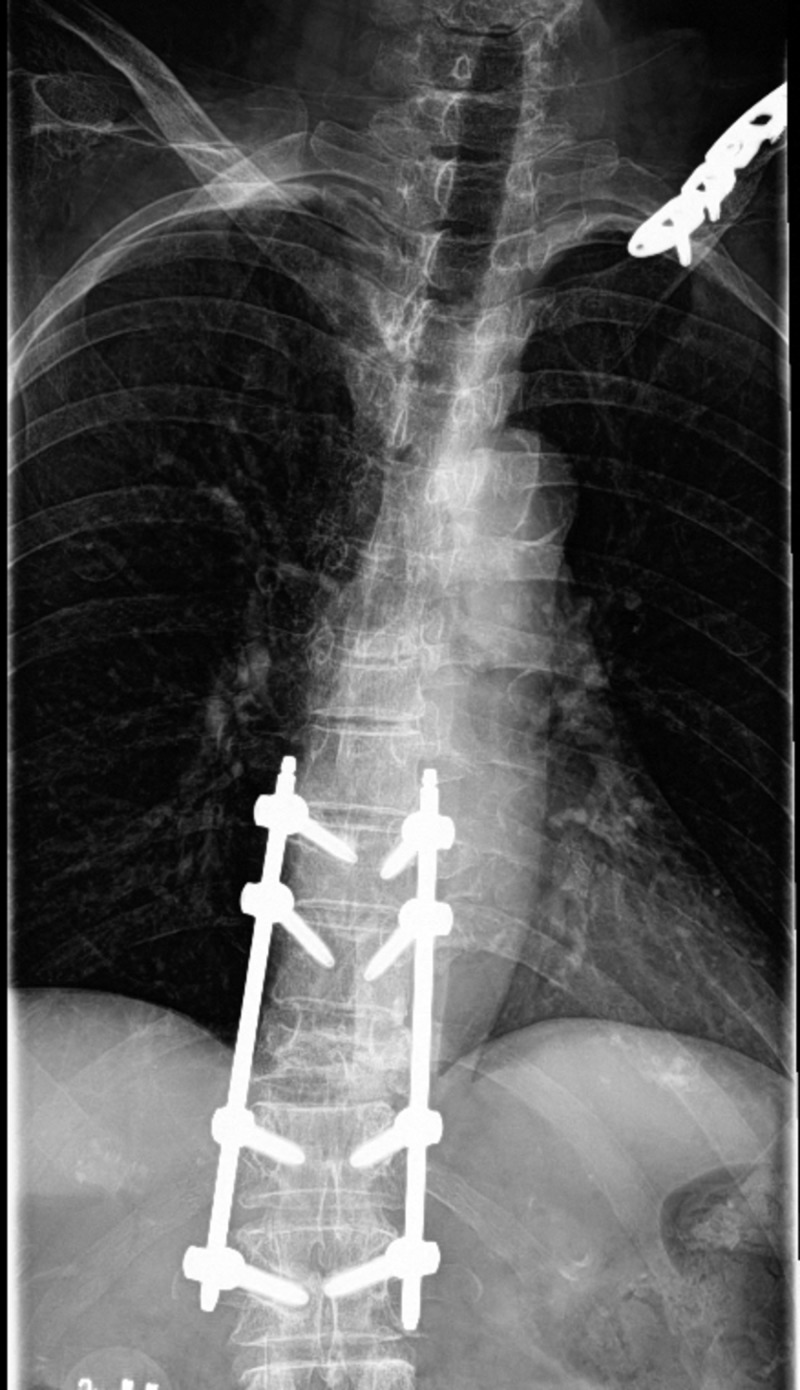
Six months post-operative thoracic spine X-ray with intact hardware without lucency, left clavicular plate

The patient’s initial post-operative course was uneventful with complete resolution of her back pain. Wounds were healing well without erythema or drainage. One month post operatively, the patient was noted to have a 4.9 kilogram weight loss, anorexia, and fatigue. She was followed by her primary provider and was treated with a high caloric diet, merinol, and followed monthly for weight management. She began to develop pain along the hardware site approximately six months post operatively which was felt to be due to weight loss and thoracic hardware near the skin's surface. The incision sites appeared to be well-healed upon examination in the clinic. Complete blood count with differential was ordered to rule out delayed infectious process and resulted within normal limits. Post-operative three months and six months X-rays demonstrated intact hardware without evidence of lucency (Figures 3-4). 

Of note, the left clavicle was initially treated conservatively. However, due to non-union, a clavicular plate was placed six weeks post spine fusion. She developed a skin rash over the clavicular plate site nine months post operatively and it was removed. At that time, the patient reported a nickel allergy she has had since childhood, which would likely explain the rash over the clavicular plate. Although there were no outward signs of skin rash along the titanium spine hardware, the patient was referred to a dermatologist for possible titanium allergy testing. According to the patient, memory lymphocyte immunostimulation assay (MELISA) testing was not performed and we were unable to obtain her dermatology clinic visit notes. She continued to decline over the next two years, ultimately losing nearly 23 kilograms. Due to the risk of skin erosion over the thoracic hardware, the hardware was removed. No caseous necrosis or metallic debris was noted surrounding the peri-prosthetic tissue during hardware removal. Therefore, no tissue samples were taken. The patient’s anorexia and fatigue improved significantly within one month of hardware removal. She gained 18 kilograms over the next six months and reported significant improvement in stamina.

## Discussion

Neurosurgical patients with a titanium hypersensitivity can be difficult to diagnose due to the rarity of the condition and non-specific presenting symptoms. Post-surgical metal hypersensitivity is more commonly associated with metal on metal total hip arthrodesis and is thought to be due to normal wear erosion causing metallic debris [1]. This debris is more commonly found in the surrounding peri-prosthetic tissue and has been described as pseudo-tumor, necrosis, and aseptic lymphocytic vasculitis associated lesions [2]. It is thought that these metallic ions form haptenic antigens with native proteins which induce a late-onset type IV hypersensitivity [3]. Clinical presentation to metallic implant hypersensitivity is generally non-specific, most frequently noted as late-onset persistent unexplained pain after an initial pain-free interval [4]. Patients with predisposed metal hypersensitivity may initiate or flare non-specific fatigue and pain syndromes with increased exposure [5]. Titanium is utilized as an alternative to nickel and cobalt chrome implants due to its inert nature within the body and low incidence of allergic reactivity [6]. Metal hypersensitivity reactions are usually manifested as a T cell-mediated delayed Type 4 reaction with characteristic cutaneous pruritic lesions [7]. Implant related metal hypersensitivity is a rare phenomenon and is most commonly associated with delayed lymphocytic reaction to metal on metal bearing surfaces in total joint arthroplasty and to a lesser extent in spinal implants [8]. Spine fixation systems are designed to be static load-bearing devices for the facilitation of successful fusion. Although considered static, these devices are subjected to micro-motion and fretting during the fusion process as well as load sharing post successful fusion which can lead to further fretting and/or hardware breakage. All of which can lead to metallic debris and subsequent hypersensitivity reaction [9]. Symptoms of implanted metal hypersensitivity can be difficult to diagnose. In orthopedic joint surgery, reported metal hypersensitivity reactions include cutaneous rash and erythema to joint pain and aseptic implant loosening [10]. Spinal fusion and disc replacement metal hypersensitivity reactions were reported as an initial pain-free interval with delayed persistent pain. Considerations high on the differential diagnosis are infection, adjunct disease, and hardware failure. Initial patient evaluation should include basic physical examination, routine laboratory testing, and imaging studies. In patients with metal hypersensitivity reactions, various imaging demonstrated peri-prosthetic tissue swelling with or without implant loosening [8-9,11]. Peri-prosthetic tissue samples consistently demonstrate a predominance of lymphocyte proliferation [8]. However, the reaction may not remain a local phenomenon. Elevated metal ion serum levels are associated with instrumented spinal fusion [12]. Metallic particles have also been located within lymph nodes, liver, and spleen [13]. Heavy and transition metals such as titanium bind to sulfur within the mitochondria inducing free radical formation triggering inflammatory, autoimmune and hypersensitivity reactions [14]. Chronic metal induced inflammation may initiate or aggravate non-specific systemic symptoms and may be attributed to auto-immune/inflammatory syndrome induced by adjuvants (ASIA) [5].

In our case, physical examination and laboratory testing were within normal limits. Radiographic imaging did not demonstrate hardware lucency. MELISA testing was not offered to this patient for reasons unknown. However, MELISA testing has a weak sensitivity to titanium and may produce false negative results [15]. Strengths of this case report include the identification of a systemic response to titanium spinal implant hypersensitivity. Spine surgeons should consider this possibility in their differential when the post-operative course includes unexplained weight-loss and fatigue. Limitations of this case study include not obtaining peri-prosthetic tissue samples and MELISA testing which may have confirmed/supported titanium hypersensitivity diagnosis. One can also not discount a psychological component to nondescript complaints and resolution of symptoms with the removal of implants.

## Conclusions

The overall incidence of titanium allergy is unknown in spinal instrumented fixation procedures. Our report offers that suspected titanium hypersensitivity, although rare in spinal instrumentation, may be linked to systemic auto-immune/inflammatory syndrome induced by metal adjuvants. Patients should be specifically queried regarding any metal hypersensitivities prior to implantation of spine hardware. Those patients with a known metal allergy should be monitored closely for local and systemic signs and symptoms pointing to hypersensitivity. We recommend removal of spinal hardware in patients with suspected systemic and local titanium hypersensitivity reaction when no other causation is identified and fixation hardware is no longer indicated or can be replaced with non-metallic devices. 
